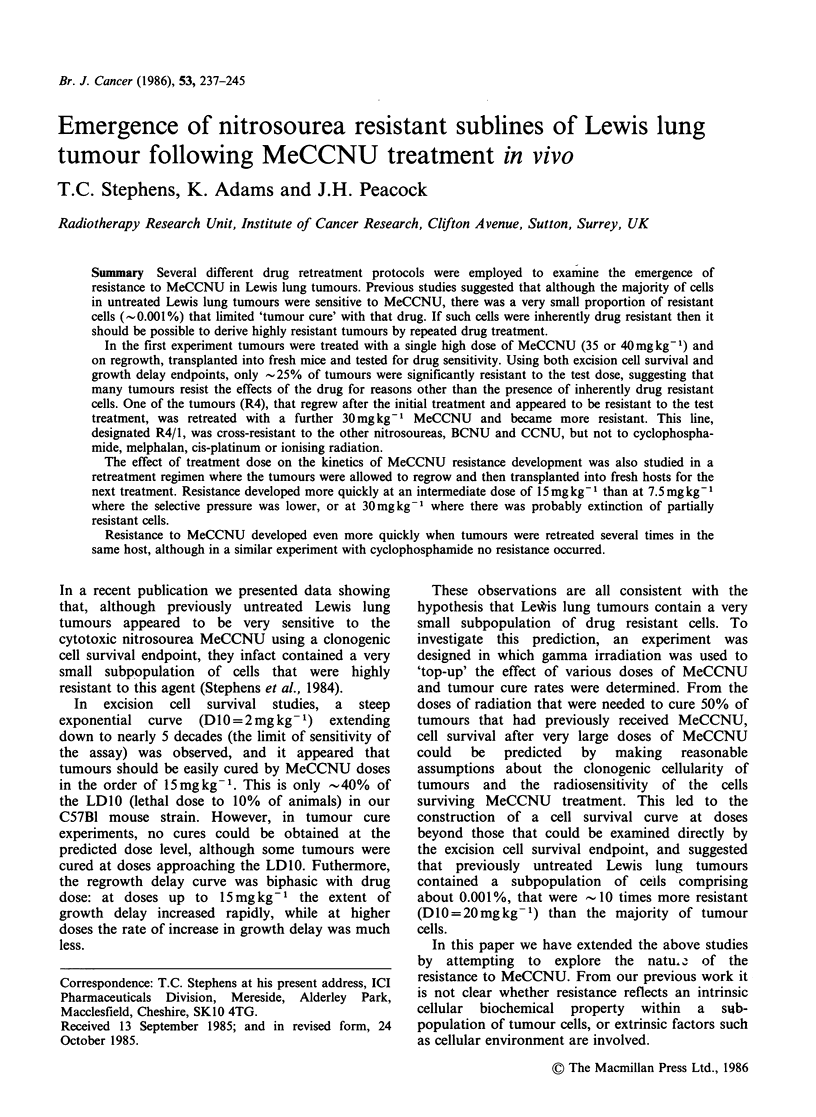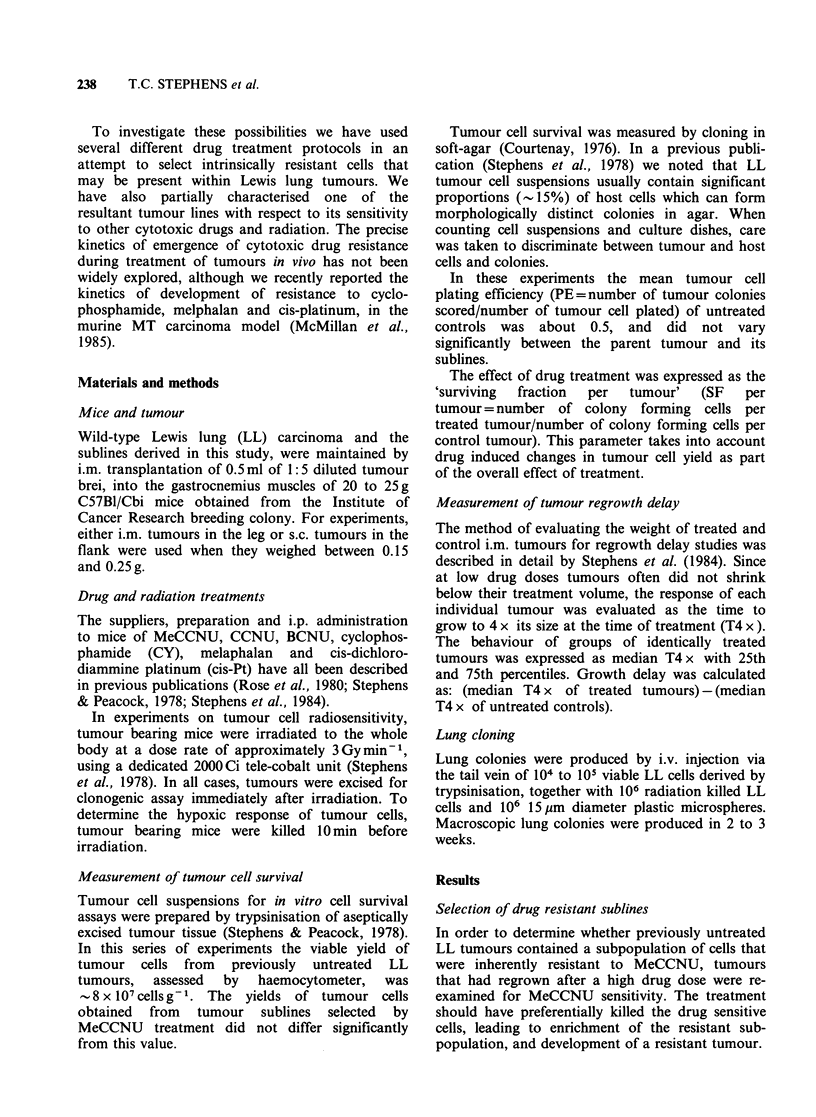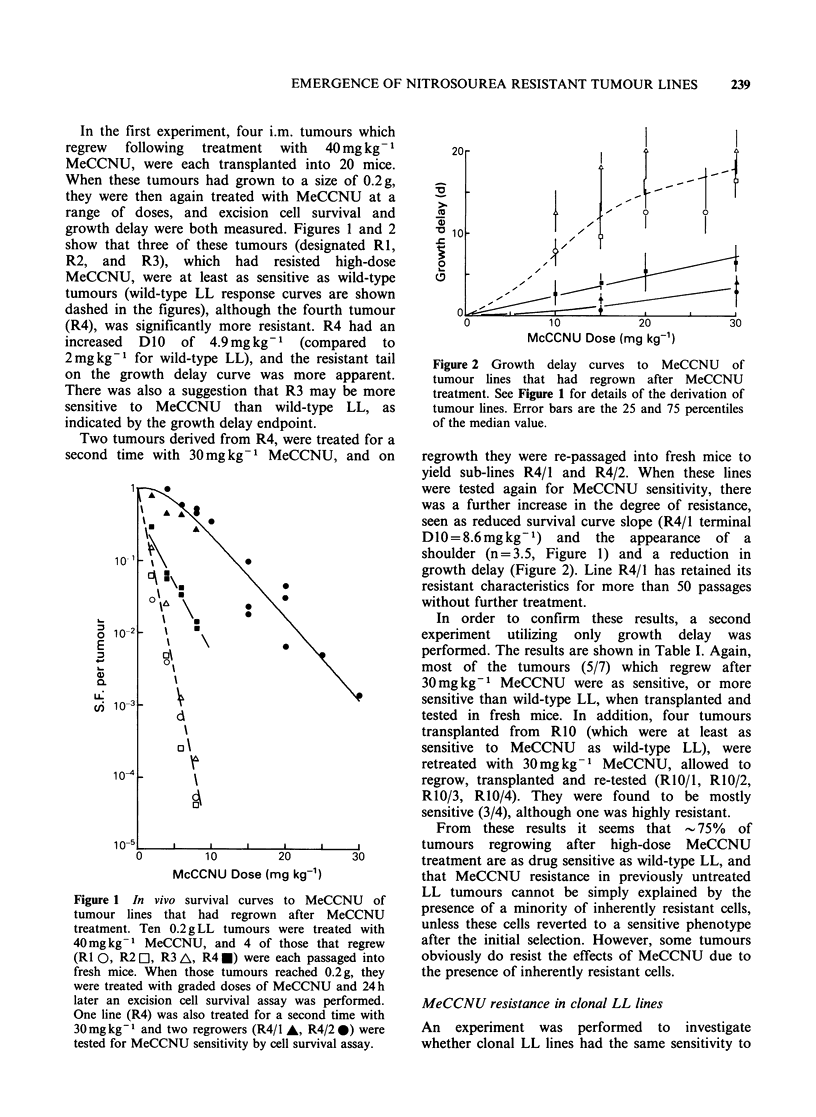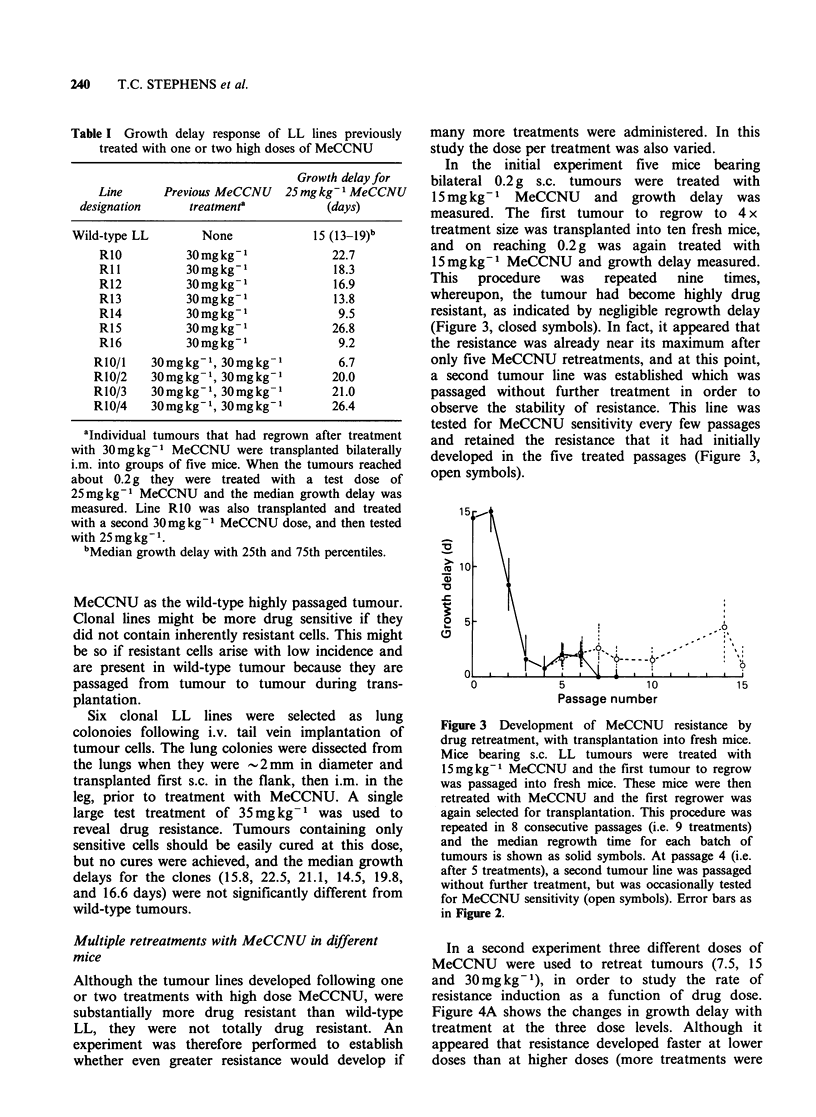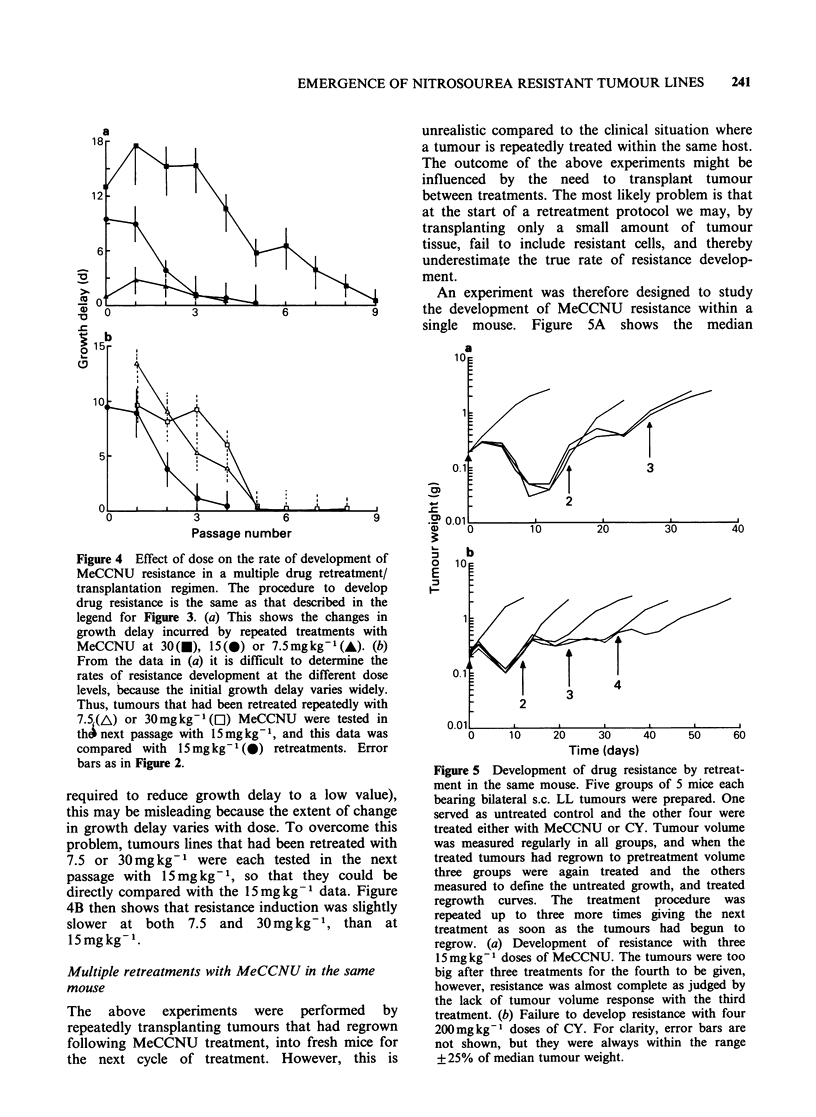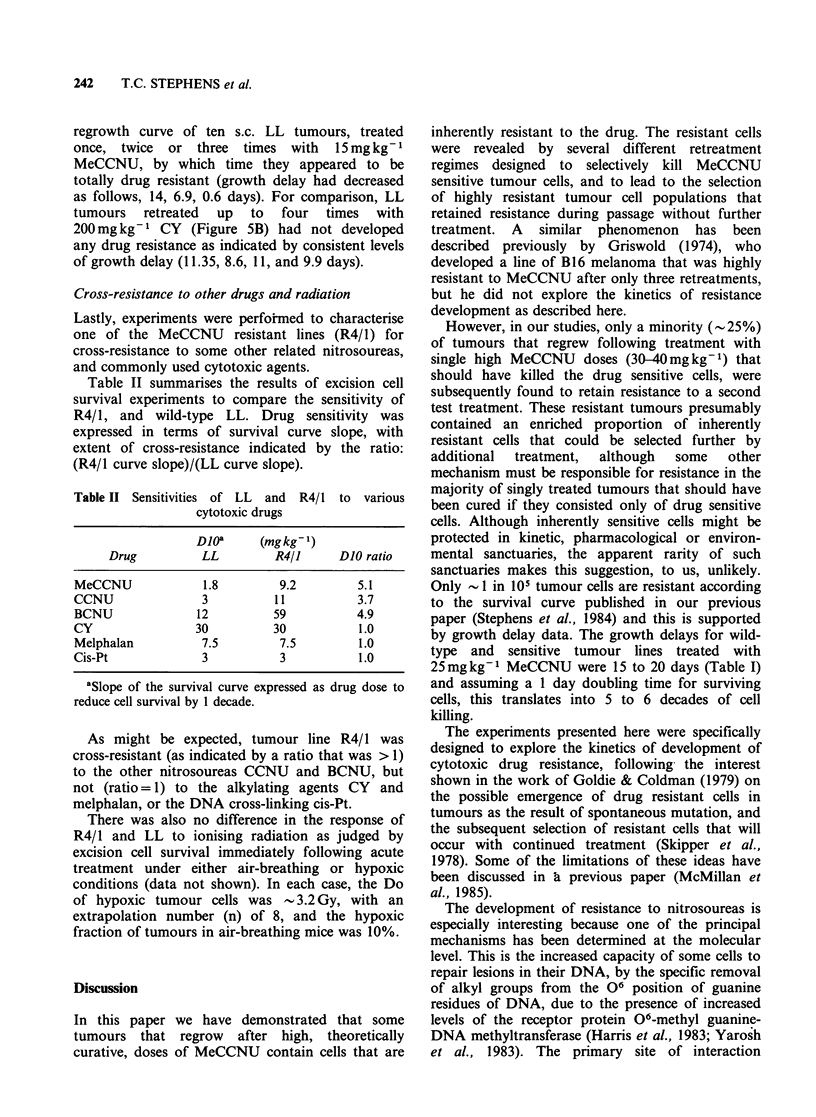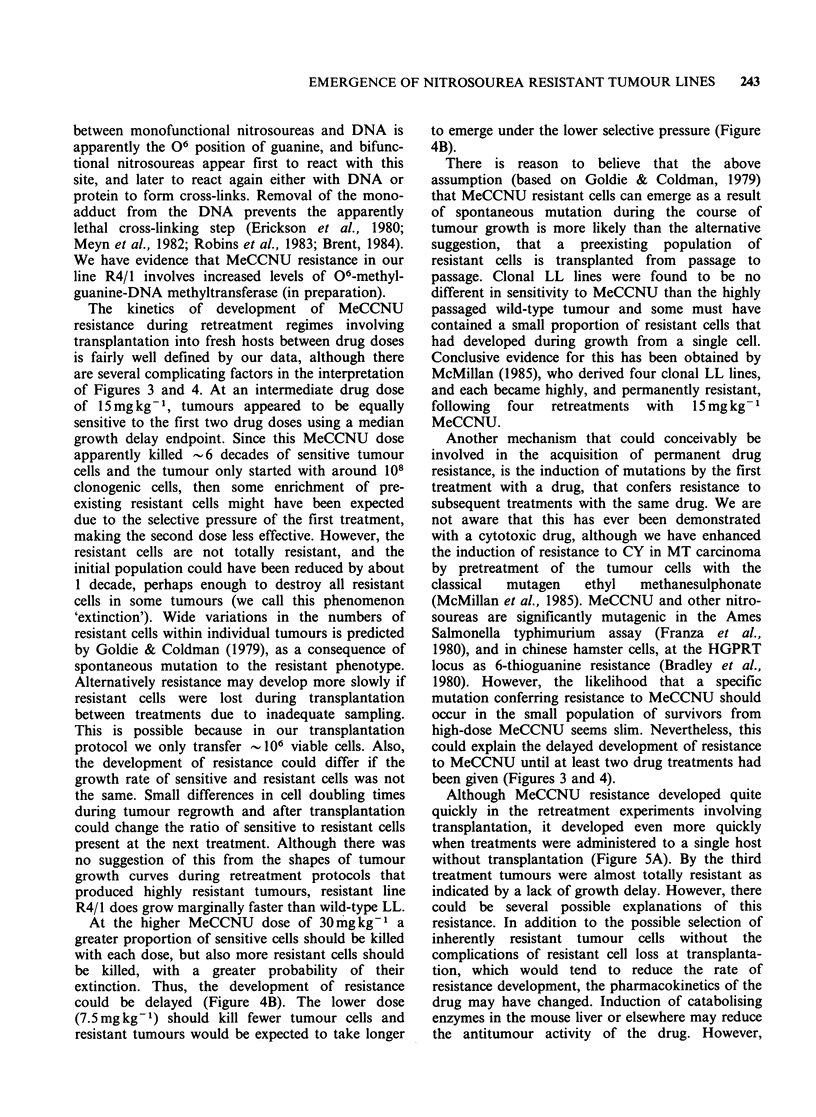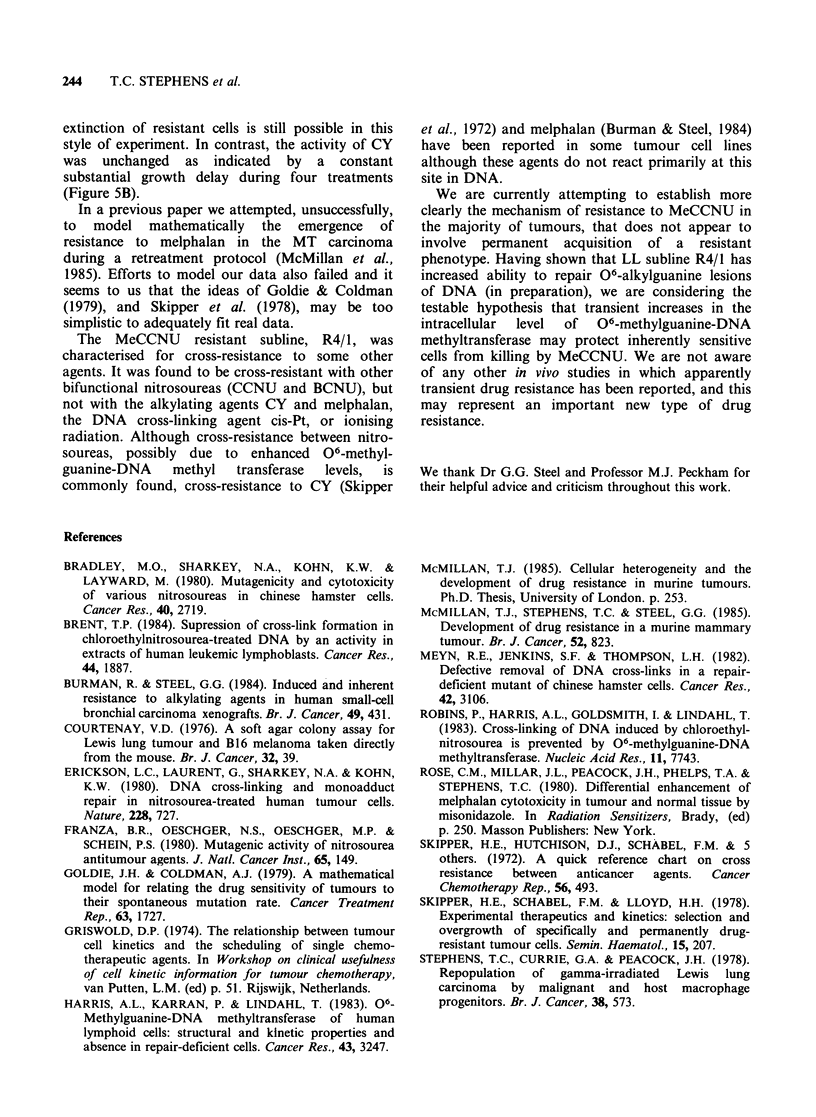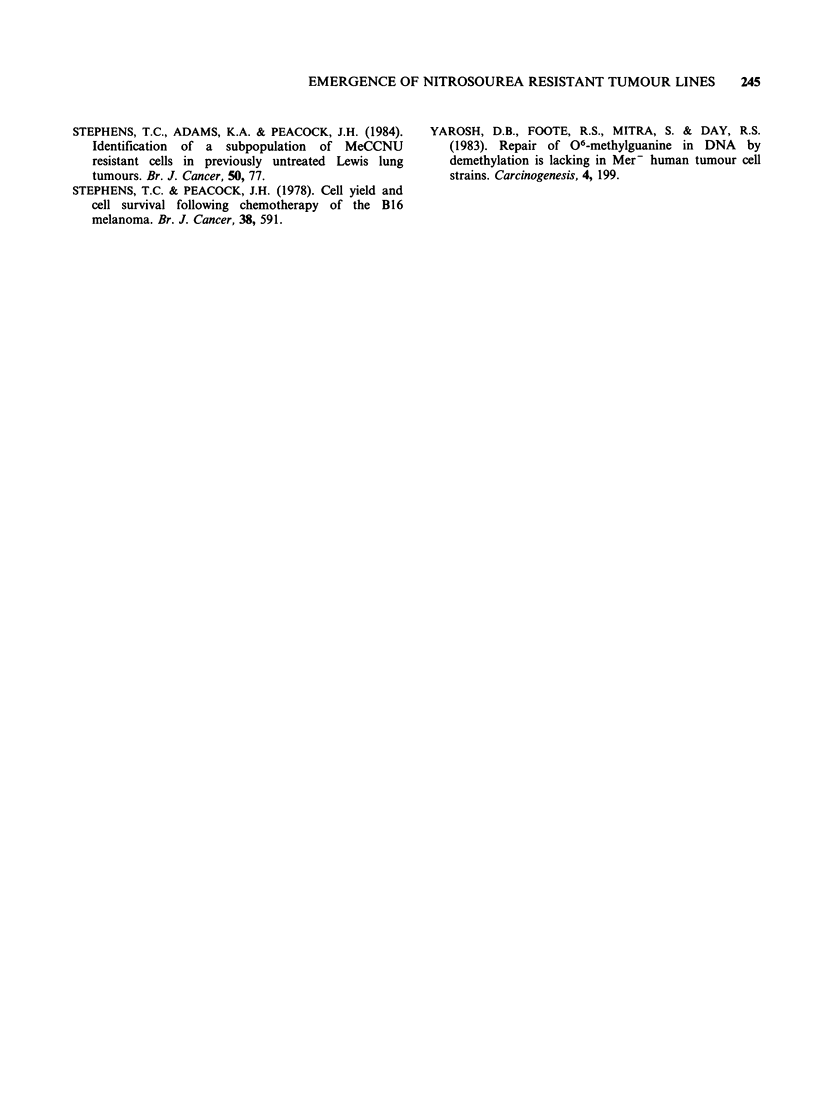# Emergence of nitrosourea resistant sublines of Lewis lung tumour following MeCCNU treatment in vivo.

**DOI:** 10.1038/bjc.1986.41

**Published:** 1986-02

**Authors:** T. C. Stephens, K. Adams, J. H. Peacock

## Abstract

Several different drug retreatment protocols were employed to examine the emergence of resistance to MeCCNU in Lewis lung tumours. Previous studies suggested that although the majority of cells in untreated Lewis lung tumours were sensitive to MeCCNU, there was a very small proportion of resistant cells (approximately 0.001%) that limited "tumour cure' with that drug. If such cells were inherently drug resistant then it should be possible to derive highly resistant tumours by repeated drug treatment. In the first experiment tumours were treated with a single high dose of MeCCNU (35 or 40 mgkg-1) and on regrowth, transplanted into fresh mice and tested for drug sensitivity. Using both excision cell survival and growth delay endpoints, only approximately 25% of tumours were significantly resistant to the test dose, suggesting that many tumours resist the effects of the drug for reasons other than the presence of inherently drug resistant cells. One of the tumours (R4), that regrew after the initial treatment and appeared to be resistant to the test treatment, was retreated with a further 30 mgkg-1 MeCCNU and became more resistant. This line, designated R4/1, was cross-resistant to the other nitrosoureas, BCNU and CCNU, but not to cyclophosphamide, melphalan, cis-platinum or ionising radiation. The effect of treatment dose on the kinetics of MeCCNU resistance development was also studied in a retreatment regimen where the tumours were allowed to regrow and then transplanted into fresh hosts for the next treatment. Resistance developed more quickly at an intermediate dose of 15 mgkg-1 than at 7.5 mgkg-1 where the selective pressure was lower, or at 30 mgkg-1 where there was probably extinction of partially resistant cells. Resistance to MeCCNU developed even more quickly when tumours were retreated several times in the same host, although in a similar experiment with cyclophosphamide no resistance occurred.


*Br. J. Cancer* (1986), **53**, 237–245

### Emergence of nitrosourea resistant sublines of Lewis lung tumour following MeCCNU treatment *in vivo*

T.C. Stephens, K. Adams and J.H. Peacock

*Radiotherapy Research Unit, Institute of Cancer Research, Clifton Avenue, Sutton, Surrey, UK*

**Summary** Several different drug retreatment protocols were employed to examine the emergence of resistance to MeCCNU in Lewis lung tumours. Previous studies suggested that although the majority of cells in untreated Lewis lung tumours were sensitive to MeCCNU, there was a very small proportion of resistant cells (~0.001%) that limited 'tumour cure' with that drug. If such cells were inherently drug resistant then it should be possible to derive highly resistant tumours by repeated drug treatment.

In the first experiment tumours were treated with a single high dose of MeCCNU (35 or 40 mg kg$^{-1}$) and on regrowth, transplanted into fresh mice and tested for drug sensitivity. Using both excision cell survival and growth delay endpoints, only ~25% of tumours were significantly resistant to the test dose, suggesting that many tumours resist the effects of the drug for reasons other than the presence of inherently drug resistant cells. One of the tumours (R4), that regrew after the initial treatment and appeared to be resistant to the test treatment, was retreated with a further 30 mg kg$^{-1}$ MeCCNU and became more resistant. This line, designated R4/1, was cross-resistant to the other nitrosoureas, BCNU and CCNU, but not to cyclophosphamide, melphalan, cis-platinum or ionising radiation.

The effect of treatment dose on the kinetics of MeCCNU resistance development was also studied in a retreatment regimen where the tumours were allowed to regrow and then transplanted into fresh hosts for the next treatment. Resistance developed more quickly at an intermediate dose of 15 mg kg$^{-1}$ than at 7.5 mg kg$^{-1}$ where the selective pressure was lower, or at 30 mg kg$^{-1}$ where there was probably extinction of partially resistant cells.

Resistance to MeCCNU developed even more quickly when tumours were retreated several times in the same host, although in a similar experiment with cyclophosphamide no resistance occurred.

In a recent publication we presented data showing that, although previously untreated Lewis lung tumours appeared to be very sensitive to the cytotoxic nitrosourea MeCCNU using a clonogenic cell survival endpoint, they infact contained a very small subpopulation of cells that were highly resistant to this agent (Stephens *et al.*, 1984).

In excision cell survival studies, a steep exponential curve (D10 = 2 mg kg$^{-1}$) extending down to nearly 5 decades (the limit of sensitivity of the assay) was observed, and it appeared that tumours should be easily cured by MeCCNU doses in the order of 15 mg kg$^{-1}$. This is only ~40% of the LD10 (lethal dose to 10% of animals) in our C57Bl mouse strain. However, in tumour cure experiments, no cures could be obtained at the predicted dose level, although some tumours were cured at doses approaching the LD10. Futhermore, the regrowth delay curve was biphasic with drug dose: at doses up to 15 mg kg$^{-1}$ the extent of growth delay increased rapidly, while at higher doses the rate of increase in growth delay was much less.

These observations are all consistent with the hypothesis that Lewis lung tumours contain a very small subpopulation of drug resistant cells. To investigate this prediction, an experiment was designed in which gamma irradiation was used to 'top-up' the effect of various doses of MeCCNU and tumour cure rates were determined. From the doses of radiation that were needed to cure 50% of tumours that had previously received MeCCNU, cell survival after very large doses of MeCCNU could be predicted by making reasonable assumptions about the clonogenic cellularity of tumours and the radiosensitivity of the cells surviving MeCCNU treatment. This led to the construction of a cell survival curve at doses beyond those that could be examined directly by the excision cell survival endpoint, and suggested that previously untreated Lewis lung tumours contained a subpopulation of cells comprising about 0.001%, that were ~10 times more resistant (D10 = 20 mg kg$^{-1}$) than the majority of tumour cells.

In this paper we have extended the above studies by attempting to explore the nature of the resistance to MeCCNU. From our previous work it is not clear whether resistance reflects an intrinsic cellular biochemical property within a subpopulation of tumour cells, or extrinsic factors such as cellular environment are involved.

Correspondence: T.C. Stephens at his present address, ICI Pharmaceuticals Division, Mereside, Alderley Park, Macclesfield, Cheshire, SK10 4TG.

Received 13 September 1985; and in revised form, 24 October 1985.

© The Macmillan Press Ltd., 1986

To investigate these possibilities we have used several different drug treatment protocols in an attempt to select intrinsically resistant cells that may be present within Lewis lung tumours. We have also partially characterised one of the resultant tumour lines with respect to its sensitivity to other cytotoxic drugs and radiation. The precise kinetics of emergence of cytotoxic drug resistance during treatment of tumours *in vivo* has not been widely explored, although we recently reported the kinetics of development of resistance to cyclophosphamide, melphalan and cis-platinum, in the murine MT carcinoma model (McMillan *et al.*, 1985).

### Materials and methods

### *Mice and tumour*

Wild-type Lewis lung (LL) carcinoma and the sublines derived in this study, were maintained by i.m. transplantation of 0.5 ml of 1:5 diluted tumour brei, into the gastrocnemius muscles of 20 to 25 g C57Bl/Cbi mice obtained from the Institute of Cancer Research breeding colony. For experiments, either i.m. tumours in the leg or s.c. tumours in the flank were used when they weighed between 0.15 and 0.25 g.

### *Drug and radiation treatments*

The suppliers, preparation and i.p. administration to mice of MeCCNU, CCNU, BCNU, cyclophosphamide (CY), melaphalan and cis-dichlorodiammine platinum (cis-Pt) have all been described in previous publications (Rose *et al.*, 1980; Stephens & Peacock, 1978; Stephens *et al.*, 1984).

In experiments on tumour cell radiosensitivity, tumour bearing mice were irradiated to the whole body at a dose rate of approximately 3 Gy min$^{-1}$, using a dedicated 2000 Ci tele-cobalt unit (Stephens *et al.*, 1978). In all cases, tumours were excised for clonogenic assay immediately after irradiation. To determine the hypoxic response of tumour cells, tumour bearing mice were killed 10 min before irradiation.

### *Measurement of tumour cell survival*

Tumour cell suspensions for *in vitro* cell survival assays were prepared by trypsinisation of aseptically excised tumour tissue (Stephens & Peacock, 1978). In this series of experiments the viable yield of tumour cells from previously untreated LL tumours, assessed by haemocytometer, was $\sim 8 \times 10^7$ cells g$^{-1}$. The yields of tumour cells obtained from tumour sublines selected by MeCCNU treatment did not differ significantly from this value.

Tumour cell survival was measured by cloning in soft-agar (Courtenay, 1976). In a previous publication (Stephens *et al.*, 1978) we noted that LL tumour cell suspensions usually contain significant proportions ($\sim$15%) of host cells which can form morphologically distinct colonies in agar. When counting cell suspensions and culture dishes, care was taken to discriminate between tumour and host cells and colonies.

In these experiments the mean tumour cell plating efficiency (PE = number of tumour colonies scored/number of tumour cell plated) of untreated controls was about 0.5, and did not vary significantly between the parent tumour and its sublines.

The effect of drug treatment was expressed as the 'surviving fraction per tumour' (SF per tumour = number of colony forming cells per treated tumour/number of colony forming cells per control tumour). This parameter takes into account drug induced changes in tumour cell yield as part of the overall effect of treatment.

### *Measurement of tumour regrowth delay*

The method of evaluating the weight of treated and control i.m. tumours for regrowth delay studies was described in detail by Stephens *et al.* (1984). Since at low drug doses tumours often did not shrink below their treatment volume, the response of each individual tumour was evaluated as the time to grow to 4 × its size at the time of treatment (T4 ×). The behaviour of groups of identically treated tumours was expressed as median T4 × with 25th and 75th percentiles. Growth delay was calculated as: (median T4 × of treated tumours) – (median T4 × of untreated controls).

### *Lung cloning*

Lung colonies were produced by i.v. injection via the tail vein of $10^4$ to $10^5$ viable LL cells derived by trypsinisation, together with $10^6$ radiation killed LL cells and $10^6$ 15 $\mu$m diameter plastic microspheres. Macroscopic lung colonies were produced in 2 to 3 weeks.

### Results

### *Selection of drug resistant sublines*

In order to determine whether previously untreated LL tumours contained a subpopulation of cells that were inherently resistant to MeCCNU, tumours that had regrown after a high drug dose were re-examined for MeCCNU sensitivity. The treatment should have preferentially killed the drug sensitive cells, leading to enrichment of the resistant subpopulation, and development of a resistant tumour.

In the first experiment, four i.m. tumours which regrew following treatment with 40 mg kg$^{-1}$ MeCCNU, were each transplanted into 20 mice. When these tumours had grown to a size of 0.2 g, they were then again treated with MeCCNU at a range of doses, and excision cell survival and growth delay were both measured. Figures 1 and 2 show that three of these tumours (designated R1, R2, and R3), which had resisted high-dose MeCCNU, were at least as sensitive as wild-type tumours (wild-type LL response curves are shown dashed in the figures), although the fourth tumour (R4), was significantly more resistant. R4 had an increased D10 of 4.9 mg kg$^{-1}$ (compared to 2 mg kg$^{-1}$ for wild-type LL), and the resistant tail on the growth delay curve was more apparent. There was also a suggestion that R3 may be more sensitive to MeCCNU than wild-type LL, as indicated by the growth delay endpoint.

Two tumours derived from R4, were treated for a second time with 30 mg kg$^{-1}$ MeCCNU, and on

**Figure 1** *In vivo* survival curves to MeCCNU of tumour lines that had regrown after MeCCNU treatment. Ten 0.2 g LL tumours were treated with 40 mg kg$^{-1}$ MeCCNU, and 4 of those that regrew (R1 ○, R2 □, R3 △, R4 ■) were each passaged into fresh mice. When those tumours reached 0.2 g, they were treated with graded doses of MeCCNU and 24 h later an excision cell survival assay was performed. One line (R4) was also treated for a second time with 30 mg kg$^{-1}$ and two regrowers (R4/1 ▲, R4/2 ●) were tested for MeCCNU sensitivity by cell survival assay.

**Figure 2** Growth delay curves to MeCCNU of tumour lines that had regrown after MeCCNU treatment. See **Figure 1** for details of the derivation of tumour lines. Error bars are the 25 and 75 percentiles of the median value.

regrowth they were re-passaged into fresh mice to yield sub-lines R4/1 and R4/2. When these lines were tested again for MeCCNU sensitivity, there was a further increase in the degree of resistance, seen as reduced survival curve slope (R4/1 terminal D10 = 8.6 mg kg$^{-1}$) and the appearance of a shoulder (n = 3.5, Figure 1) and a reduction in growth delay (Figure 2). Line R4/1 has retained its resistant characteristics for more than 50 passages without further treatment.

In order to confirm these results, a second experiment utilizing only growth delay was performed. The results are shown in Table I. Again, most of the tumours (5/7) which regrew after 30 mg kg$^{-1}$ MeCCNU were as sensitive, or more sensitive than wild-type LL, when transplanted and tested in fresh mice. In addition, four tumours transplanted from R10 (which were at least as sensitive to MeCCNU as wild-type LL), were retreated with 30 mg kg$^{-1}$ MeCCNU, allowed to regrow, transplanted and re-tested (R10/1, R10/2, R10/3, R10/4). They were found to be mostly sensitive (3/4), although one was highly resistant.

From these results it seems that ~75% of tumours regrowing after high-dose MeCCNU treatment are as drug sensitive as wild-type LL, and that MeCCNU resistance in previously untreated LL tumours cannot be simply explained by the presence of a minority of inherently resistant cells, unless these cells reverted to a sensitive phenotype after the initial selection. However, some tumours obviously do resist the effects of MeCCNU due to the presence of inherently resistant cells.

### *MeCCNU resistance in clonal LL lines*

An experiment was performed to investigate whether clonal LL lines had the same sensitivity to

**Table I** Growth delay response of LL lines previously treated with one or two high doses of MeCCNU

| *Line designation* | *Previous MeCCNU treatment*[a] | *Growth delay for 25 mg kg$^{-1}$ MeCCNU (days)* |
|---|---|---|
| Wild-type LL | None | 15 (13–19)[b] |
| R10 | 30 mg kg$^{-1}$ | 22.7 |
| R11 | 30 mg kg$^{-1}$ | 18.3 |
| R12 | 30 mg kg$^{-1}$ | 16.9 |
| R13 | 30 mg kg$^{-1}$ | 13.8 |
| R14 | 30 mg kg$^{-1}$ | 9.5 |
| R15 | 30 mg kg$^{-1}$ | 26.8 |
| R16 | 30 mg kg$^{-1}$ | 9.2 |
| R10/1 | 30 mg kg$^{-1}$, 30 mg kg$^{-1}$ | 6.7 |
| R10/2 | 30 mg kg$^{-1}$, 30 mg kg$^{-1}$ | 20.0 |
| R10/3 | 30 mg kg$^{-1}$, 30 mg kg$^{-1}$ | 21.0 |
| R10/4 | 30 mg kg$^{-1}$, 30 mg kg$^{-1}$ | 26.4 |

[a]Individual tumours that had regrown after treatment with 30 mg kg$^{-1}$ MeCCNU were transplanted bilaterally i.m. into groups of five mice. When the tumours reached about 0.2 g they were treated with a test dose of 25 mg kg$^{-1}$ MeCCNU and the median growth delay was measured. Line R10 was also transplanted and treated with a second 30 mg kg$^{-1}$ MeCCNU dose, and then tested with 25 mg kg$^{-1}$.
[b]Median growth delay with 25th and 75th percentiles.

MeCCNU as the wild-type highly passaged tumour. Clonal lines might be more drug sensitive if they did not contain inherently resistant cells. This might be so if resistant cells arise with low incidence and are present in wild-type tumour because they are passaged from tumour to tumour during transplantation.

Six clonal LL lines were selected as lung colonoies following i.v. tail vein implantation of tumour cells. The lung colonies were dissected from the lungs when they were ~2 mm in diameter and transplanted first s.c. in the flank, then i.m. in the leg, prior to treatment with MeCCNU. A single large test treatment of 35 mg kg$^{-1}$ was used to reveal drug resistance. Tumours containing only sensitive cells should be easily cured at this dose, but no cures were achieved, and the median growth delays for the clones (15.8, 22.5, 21.1, 14.5, 19.8, and 16.6 days) were not significantly different from wild-type tumours.

### *Multiple retreatments with MeCCNU in different mice*

Although the tumour lines developed following one or two treatments with high dose MeCCNU, were substantially more drug resistant than wild-type LL, they were not totally drug resistant. An experiment was therefore performed to establish whether even greater resistance would develop if many more treatments were administered. In this study the dose per treatment was also varied.

In the initial experiment five mice bearing bilateral 0.2 g s.c. tumours were treated with 15 mg kg$^{-1}$ MeCCNU and growth delay was measured. The first tumour to regrow to 4× treatment size was transplanted into ten fresh mice, and on reaching 0.2 g was again treated with 15 mg kg$^{-1}$ MeCCNU and growth delay measured. This procedure was repeated nine times, whereupon, the tumour had become highly drug resistant, as indicated by negligible regrowth delay (Figure 3, closed symbols). In fact, it appeared that the resistance was already near its maximum after only five MeCCNU retreatments, and at this point, a second tumour line was established which was passaged without further treatment in order to observe the stability of resistance. This line was tested for MeCCNU sensitivity every few passages and retained the resistance that it had initially developed in the five treated passages (Figure 3, open symbols).

**Figure 3** Development of MeCCNU resistance by drug retreatment, with transplantation into fresh mice. Mice bearing s.c. LL tumours were treated with 15 mg kg$^{-1}$ MeCCNU and the first tumour to regrow was passaged into fresh mice. These mice were then retreated with MeCCNU and the first regrower was again selected for transplantation. This procedure was repeated in 8 consecutive passages (i.e. 9 treatments) and the median regrowth time for each batch of tumours is shown as solid symbols. At passage 4 (i.e. after 5 treatments), a second tumour line was passaged without further treatment, but was occasionally tested for MeCCNU sensitivity (open symbols). Error bars as in **Figure 2**.

In a second experiment three different doses of MeCCNU were used to retreat tumours (7.5, 15 and 30 mg kg$^{-1}$), in order to study the rate of resistance induction as a function of drug dose. Figure 4A shows the changes in growth delay with treatment at the three dose levels. Although it appeared that resistance developed faster at lower doses than at higher doses (more treatments were

**Figure 4** Effect of dose on the rate of development of MeCCNU resistance in a multiple drug retreatment/transplantation regimen. The procedure to develop drug resistance is the same as that described in the legend for **Figure** 3. (*a*) This shows the changes in growth delay incurred by repeated treatments with MeCCNU at 30(■), 15(●) or 7.5 mg kg$^{-1}$(▲). (*b*) From the data in (*a*) it is difficult to determine the rates of resistance development at the different dose levels, because the initial growth delay varies widely. Thus, tumours that had been retreated repeatedly with 7.5 (△) or 30 mg kg$^{-1}$(□) MeCCNU were tested in the next passage with 15 mg kg$^{-1}$, and this data was compared with 15 mg kg$^{-1}$(●) retreatments. Error bars as in **Figure 2**.

required to reduce growth delay to a low value), this may be misleading because the extent of change in growth delay varies with dose. To overcome this problem, tumours lines that had been retreated with 7.5 or 30 mg kg$^{-1}$ were each tested in the next passage with 15 mg kg$^{-1}$, so that they could be directly compared with the 15 mg kg$^{-1}$ data. Figure 4B then shows that resistance induction was slightly slower at both 7.5 and 30 mg kg$^{-1}$, than at 15 mg kg$^{-1}$.

### *Multiple retreatments with MeCCNU in the same mouse*

The above experiments were performed by repeatedly transplanting tumours that had regrown following MeCCNU treatment, into fresh mice for the next cycle of treatment. However, this is unrealistic compared to the clinical situation where a tumour is repeatedly treated within the same host. The outcome of the above experiments might be influenced by the need to transplant tumour between treatments. The most likely problem is that at the start of a retreatment protocol we may, by transplanting only a small amount of tumour tissue, fail to include resistant cells, and thereby underestimate the true rate of resistance development.

An experiment was therefore designed to study the development of MeCCNU resistance within a single mouse. Figure 5A shows the median

**Figure 5** Development of drug resistance by retreatment in the same mouse. Five groups of 5 mice each bearing bilateral s.c. LL tumours were prepared. One served as untreated control and the other four were treated either with MeCCNU or CY. Tumour volume was measured regularly in all groups, and when the treated tumours had regrown to pretreatment volume three groups were again treated and the others measured to define the untreated growth, and treated regrowth curves. The treatment procedure was repeated up to three more times giving the next treatment as soon as the tumours had begun to regrow. (*a*) Development of resistance with three 15 mg kg$^{-1}$ doses of MeCCNU. The tumours were too big after three treatments for the fourth to be given, however, resistance was almost complete as judged by the lack of tumour volume response with the third treatment. (*b*) Failure to develop resistance with four 200 mg kg$^{-1}$ doses of CY. For clarity, error bars are not shown, but they were always within the range ±25% of median tumour weight.

regrowth curve of ten s.c. LL tumours, treated once, twice or three times with 15 mg kg$^{-1}$ MeCCNU, by which time they appeared to be totally drug resistant (growth delay had decreased as follows, 14, 6.9, 0.6 days). For comparison, LL tumours retreated up to four times with 200 mg kg$^{-1}$ CY (Figure 5B) had not developed any drug resistance as indicated by consistent levels of growth delay (11.35, 8.6, 11, and 9.9 days).

*Cross-resistance to other drugs and radiation*

Lastly, experiments were performed to characterise one of the MeCCNU resistant lines (R4/1) for cross-resistance to some other related nitrosoureas, and commonly used cytotoxic agents.

Table II summarises the results of excision cell survival experiments to compare the sensitivity of R4/1, and wild-type LL. Drug sensitivity was expressed in terms of survival curve slope, with extent of cross-resistance indicated by the ratio: (R4/1 curve slope)/(LL curve slope).

**Table II** Sensitivities of LL and R4/1 to various cytotoxic drugs

| Drug | D10[a] LL | (mg kg$^{-1}$) R4/1 | D10 ratio |
|---|---|---|---|
| MeCCNU | 1.8 | 9.2 | 5.1 |
| CCNU | 3 | 11 | 3.7 |
| BCNU | 12 | 59 | 4.9 |
| CY | 30 | 30 | 1.0 |
| Melphalan | 7.5 | 7.5 | 1.0 |
| Cis-Pt | 3 | 3 | 1.0 |

[a]Slope of the survival curve expressed as drug dose to reduce cell survival by 1 decade.

As might be expected, tumour line R4/1 was cross-resistant (as indicated by a ratio that was >1) to the other nitrosoureas CCNU and BCNU, but not (ratio = 1) to the alkylating agents CY and melphalan, or the DNA cross-linking cis-Pt.

There was also no difference in the response of R4/1 and LL to ionising radiation as judged by excision cell survival immediately following acute treatment under either air-breathing or hypoxic conditions (data not shown). In each case, the Do of hypoxic tumour cells was ~3.2 Gy, with an extrapolation number (n) of 8, and the hypoxic fraction of tumours in air-breathing mice was 10%.

### Discussion

In this paper we have demonstrated that some tumours that regrow after high, theoretically curative, doses of MeCCNU contain cells that are inherently resistant to the drug. The resistant cells were revealed by several different retreatment regimes designed to selectively kill MeCCNU sensitive tumour cells, and to lead to the selection of highly resistant tumour cell populations that retained resistance during passage without further treatment. A similar phenomenon has been described previously by Griswold (1974), who developed a line of B16 melanoma that was highly resistant to MeCCNU after only three retreatments, but he did not explore the kinetics of resistance development as described here.

However, in our studies, only a minority (~25%) of tumours that regrew following treatment with single high MeCCNU doses (30–40 mg kg$^{-1}$) that should have killed the drug sensitive cells, were subsequently found to retain resistance to a second test treatment. These resistant tumours presumably contained an enriched proportion of inherently resistant cells that could be selected further by additional treatment, although some other mechanism must be responsible for resistance in the majority of singly treated tumours that should have been cured if they consisted only of drug sensitive cells. Although inherently sensitive cells might be protected in kinetic, pharmacological or environmental sanctuaries, the apparent rarity of such sanctuaries makes this suggestion, to us, unlikely. Only ~1 in $10^5$ tumour cells are resistant according to the survival curve published in our previous paper (Stephens *et al.*, 1984) and this is supported by growth delay data. The growth delays for wild-type and sensitive tumour lines treated with 25 mg kg$^{-1}$ MeCCNU were 15 to 20 days (Table I) and assuming a 1 day doubling time for surviving cells, this translates into 5 to 6 decades of cell killing.

The experiments presented here were specifically designed to explore the kinetics of development of cytotoxic drug resistance, following the interest shown in the work of Goldie & Coldman (1979) on the possible emergence of drug resistant cells in tumours as the result of spontaneous mutation, and the subsequent selection of resistant cells that will occur with continued treatment (Skipper *et al.*, 1978). Some of the limitations of these ideas have been discussed in a previous paper (McMillan *et al.*, 1985).

The development of resistance to nitrosoureas is especially interesting because one of the principal mechanisms has been determined at the molecular level. This is the increased capacity of some cells to repair lesions in their DNA, by the specific removal of alkyl groups from the $O^6$ position of guanine residues of DNA, due to the presence of increased levels of the receptor protein $O^6$-methyl guanine-DNA methyltransferase (Harris *et al.*, 1983; Yarosh *et al.*, 1983). The primary site of interaction between monofunctional nitrosoureas and DNA is apparently the $O^6$ position of guanine, and bifunctional nitrosoureas appear first to react with this site, and later to react again either with DNA or protein to form cross-links. Removal of the mono-adduct from the DNA prevents the apparently lethal cross-linking step (Erickson *et al.*, 1980; Meyn *et al.*, 1982; Robins *et al.*, 1983; Brent, 1984). We have evidence that MeCCNU resistance in our line R4/1 involves increased levels of $O^6$-methylguanine-DNA methyltransferase (in preparation).

The kinetics of development of MeCCNU resistance during retreatment regimes involving transplantation into fresh hosts between drug doses is fairly well defined by our data, although there are several complicating factors in the interpretation of Figures 3 and 4. At an intermediate drug dose of $15\,mg\,kg^{-1}$, tumours appeared to be equally sensitive to the first two drug doses using a median growth delay endpoint. Since this MeCCNU dose apparently killed $\sim 6$ decades of sensitive tumour cells and the tumour only started with around $10^8$ clonogenic cells, then some enrichment of pre-existing resistant cells might have been expected due to the selective pressure of the first treatment, making the second dose less effective. However, the resistant cells are not totally resistant, and the initial population could have been reduced by about 1 decade, perhaps enough to destroy all resistant cells in some tumours (we call this phenomenon 'extinction'). Wide variations in the numbers of resistant cells within individual tumours is predicted by Goldie & Coldman (1979), as a consequence of spontaneous mutation to the resistant phenotype. Alternatively resistance may develop more slowly if resistant cells were lost during transplantation between treatments due to inadequate sampling. This is possible because in our transplantation protocol we only transfer $\sim 10^6$ viable cells. Also, the development of resistance could differ if the growth rate of sensitive and resistant cells was not the same. Small differences in cell doubling times during tumour regrowth and after transplantation could change the ratio of sensitive to resistant cells present at the next treatment. Although there was no suggestion of this from the shapes of tumour growth curves during retreatment protocols that produced highly resistant tumours, resistant line R4/1 does grow marginally faster than wild-type LL.

At the higher MeCCNU dose of $30\,mg\,kg^{-1}$ a greater proportion of sensitive cells should be killed with each dose, but also more resistant cells should be killed, with a greater probability of their extinction. Thus, the development of resistance could be delayed (Figure 4B). The lower dose ($7.5\,mg\,kg^{-1}$) should kill fewer tumour cells and resistant tumours would be expected to take longer to emerge under the lower selective pressure (Figure 4B).

There is reason to believe that the above assumption (based on Goldie & Coldman, 1979) that MeCCNU resistant cells can emerge as a result of spontaneous mutation during the course of tumour growth is more likely than the alternative suggestion, that a preexisting population of resistant cells is transplanted from passage to passage. Clonal LL lines were found to be no different in sensitivity to MeCCNU than the highly passaged wild-type tumour and some must have contained a small proportion of resistant cells that had developed during growth from a single cell. Conclusive evidence for this has been obtained by McMillan (1985), who derived four clonal LL lines, and each became highly, and permanently resistant, following four retreatments with $15\,mg\,kg^{-1}$ MeCCNU.

Another mechanism that could conceivably be involved in the acquisition of permanent drug resistance, is the induction of mutations by the first treatment with a drug, that confers resistance to subsequent treatments with the same drug. We are not aware that this has ever been demonstrated with a cytotoxic drug, although we have enhanced the induction of resistance to CY in MT carcinoma by pretreatment of the tumour cells with the classical mutagen ethyl methanesulphonate (McMillan *et al.*, 1985). MeCCNU and other nitrosoureas are significantly mutagenic in the Ames Salmonella typhimurium assay (Franza *et al.*, 1980), and in chinese hamster cells, at the HGPRT locus as 6-thioguanine resistance (Bradley *et al.*, 1980). However, the likelihood that a specific mutation conferring resistance to MeCCNU should occur in the small population of survivors from high-dose MeCCNU seems slim. Nevertheless, this could explain the delayed development of resistance to MeCCNU until at least two drug treatments had been given (Figures 3 and 4).

Although MeCCNU resistance developed quite quickly in the retreatment experiments involving transplantation, it developed even more quickly when treatments were administered to a single host without transplantation (Figure 5A). By the third treatment tumours were almost totally resistant as indicated by a lack of growth delay. However, there could be several possible explanations of this resistance. In addition to the possible selection of inherently resistant tumour cells without the complications of resistant cell loss at transplantation, which would tend to reduce the rate of resistance development, the pharmacokinetics of the drug may have changed. Induction of catabolising enzymes in the mouse liver or elsewhere may reduce the antitumour activity of the drug. However, extinction of resistant cells is still possible in this style of experiment. In contrast, the activity of CY was unchanged as indicated by a constant substantial growth delay during four treatments (Figure 5B).

In a previous paper we attempted, unsuccessfully, to model mathematically the emergence of resistance to melphalan in the MT carcinoma during a retreatment protocol (McMillan *et al.*, 1985). Efforts to model our data also failed and it seems to us that the ideas of Goldie & Coldman (1979), and Skipper *et al.* (1978), may be too simplistic to adequately fit real data.

The MeCCNU resistant subline, R4/1, was characterised for cross-resistance to some other agents. It was found to be cross-resistant with other bifunctional nitrosoureas (CCNU and BCNU), but not with the alkylating agents CY and melphalan, the DNA cross-linking agent cis-Pt, or ionising radiation. Although cross-resistance between nitrosoureas, possibly due to enhanced $O^6$-methylguanine-DNA methyl transferase levels, is commonly found, cross-resistance to CY (Skipper *et al.*, 1972) and melphalan (Burman & Steel, 1984) have been reported in some tumour cell lines although these agents do not react primarily at this site in DNA.

We are currently attempting to establish more clearly the mechanism of resistance to MeCCNU in the majority of tumours, that does not appear to involve permanent acquisition of a resistant phenotype. Having shown that LL subline R4/1 has increased ability to repair $O^6$-alkylguanine lesions of DNA (in preparation), we are considering the testable hypothesis that transient increases in the intracellular level of $O^6$-methylguanine-DNA methyltransferase may protect inherently sensitive cells from killing by MeCCNU. We are not aware of any other *in vivo* studies in which apparently transient drug resistance has been reported, and this may represent an important new type of drug resistance.

We thank Dr G.G. Steel and Professor M.J. Peckham for their helpful advice and criticism throughout this work.

### References

BRADLEY, M.O., SHARKEY, N.A., KOHN, K.W. & LAYWARD, M. (1980). Mutagenicity and cytotoxicity of various nitrosoureas in chinese hamster cells. *Cancer Res.*, **40**, 2719.

BRENT, T.P. (1984). Supression of cross-link formation in chloroethylnitrosourea-treated DNA by an activity in extracts of human leukemic lymphoblasts. *Cancer Res.*, **44**, 1887.

BURMAN, R. & STEEL, G.G. (1984). Induced and inherent resistance to alkylating agents in human small-cell bronchial carcinoma xenografts. *Br. J. Cancer*, **49**, 431.

COURTENAY, V.D. (1976). A soft agar colony assay for Lewis lung tumour and B16 melanoma taken directly from the mouse. *Br. J. Cancer*, **32**, 39.

ERICKSON, L.C., LAURENT, G., SHARKEY, N.A. & KOHN, K.W. (1980). DNA cross-linking and monoadduct repair in nitrosourea-treated human tumour cells. *Nature*, **228**, 727.

FRANZA, B.R., OESCHGER, N.S., OESCHGER, M.P. & SCHEIN, P.S. (1980). Mutagenic activity of nitrosourea antitumour agents. *J. Natl. Cancer Inst.*, **65**, 149.

GOLDIE, J.H. & COLDMAN, A.J. (1979). A mathematical model for relating the drug sensitivity of tumours to their spontaneous mutation rate. *Cancer Treatment Rep.*, **63**, 1727.

GRISWOLD, D.P. (1974). The relationship between tumour cell kinetics and the scheduling of single chemotherapeutic agents. In *Workshop on clinical usefulness of cell kinetic information for tumour chemotherapy*, van Putten, L.M. (ed) p. 51. Rijswijk, Netherlands.

HARRIS, A.L., KARRAN, P. & LINDAHL, T. (1983). $O^6$-Methylguanine-DNA methyltransferase of human lymphoid cells: structural and kinetic properties and absence in repair-deficient cells. *Cancer Res.*, **43**, 3247.

McMILLAN, T.J. (1985). Cellular heterogeneity and the development of drug resistance in murine tumours. Ph.D. Thesis, University of London. p. 253.

McMILLAN, T.J., STEPHENS, T.C. & STEEL, G.G. (1985). Development of drug resistance in a murine mammary tumour. *Br. J. Cancer*, **52**, 823.

MEYN, R.E., JENKINS, S.F. & THOMPSON, L.H. (1982). Defective removal of DNA cross-links in a repair-deficient mutant of chinese hamster cells. *Cancer Res.*, **42**, 3106.

ROBINS, P., HARRIS, A.L., GOLDSMITH, I. & LINDAHL, T. (1983). Cross-linking of DNA induced by chloroethylnitrosourea is prevented by $O^6$-methylguanine-DNA methyltransferase. *Nucleic Acid Res.*, **11**, 7743.

ROSE, C.M., MILLAR, J.L., PEACOCK, J.H., PHELPS, T.A. & STEPHENS, T.C. (1980). Differential enhancement of melphalan cytotoxicity in tumour and normal tissue by misonidazole. In *Radiation Sensitizers*, Brady, (ed) p. 250. Masson Publishers: New York.

SKIPPER, H.E., HUTCHISON, D.J., SCHÀBEL, F.M. & 5 others. (1972). A quick reference chart on cross resistance between anticancer agents. *Cancer Chemotherapy Rep.*, **56**, 493.

SKIPPER, H.E., SCHABEL, F.M. & LLOYD, H.H. (1978). Experimental therapeutics and kinetics: selection and overgrowth of specifically and permanently drug-resistant tumour cells. *Semin. Haematol.*, **15**, 207.

STEPHENS, T.C., CURRIE, G.A. & PEACOCK, J.H. (1978). Repopulation of gamma-irradiated Lewis lung carcinoma by malignant and host macrophage progenitors. *Br. J. Cancer*, **38**, 573.

STEPHENS, T.C., ADAMS, K.A. & PEACOCK, J.H. (1984). Identification of a subpopulation of MeCCNU resistant cells in previously untreated Lewis lung tumours. *Br. J. Cancer*, **50**, 77.

STEPHENS, T.C. & PEACOCK, J.H. (1978). Cell yield and cell survival following chemotherapy of the B16 melanoma. *Br. J. Cancer*, **38**, 591.

YAROSH, D.B., FOOTE, R.S., MITRA, S. & DAY, R.S. (1983). Repair of $O^6$-methylguanine in DNA by demethylation is lacking in $Mer^-$ human tumour cell strains. *Carcinogenesis*, **4**, 199.